# Dismissed Psychiatry Residents Who Appeal: Exploring Unprofessional Behavior

**DOI:** 10.1007/s40596-023-01746-0

**Published:** 2023-02-28

**Authors:** Judith Godschalx, Walther van Mook

**Affiliations:** 1 Flevoziekenhuis Almere GGZ Centraal Flevoland, Almere, The Netherlands; 2 Maastricht University Medical Centre+, Maastricht, The Netherlands

**Keywords:** Psychiatry residency program, Dismissal, Professional competency, Ethics

## Abstract

**Objective:**

Dutch psychiatry residents who are dismissed from their training program have the opportunity to appeal the decision. Those appeals are publicly available. This report explores the appeals of residents dismissed for unprofessional behavior.

**Methods:**

The authors analyzed caselaw of dismissed psychiatry residents brought before the conciliation board of The Royal Dutch Medical Association and compared them to a control group of caselaw of dismissed family medicine residents.

**Results:**

From 2011 to 2020, 19 psychiatry residents were dismissed for failing to meet the competencies of the CanMEDS professional domain and matched with 19 family medicine residents. Most psychiatry residents deficient in professionalism were considered deficient in their attitude, in reliability of keeping agreements, or in their ability to profit from supervisors’ feedback. Insufficient professional behavior overlapped with insufficient communication, collaboration, and management. Half of the psychiatry residents with deficits in professionalism went on sick leave at some time. Between residents in psychiatry and family medicine, or between psychiatry residents with and without a favorable conciliation board decision, no statistical differences were found regarding gender, year of residency, and number of insufficient competencies.

**Conclusions:**

The deficiencies in the professionalism of residents who challenged their program director’s decision to be dismissed mostly consisted of inadequate attitude or the inability to profit from feedback, suggesting that these residents lack empathy, introspection, or both.

Psychiatrists were overrepresented in the classical Papadakis study [1] showing that unprofessional behavior in medical school is associated with unprofessional behavior in the future. Unprofessional behavior in residency is associated with an increased risk of disciplinary board measures after graduation and is an important indication for resident remediation or dismissal [2–5]. However, there is no empirical research conducted after the Papadakis study [1] specifically toward unprofessional behavior in psychiatry residents. Residents with a structural pattern of unprofessional behavior [6] fall short in the CanMEDS professional domain. The CanMEDS competency domains [7] were implemented in psychiatry residency in the Netherlands in 2009 [8, 9]. The professional domain is particularly difficult to assess and to examine [10], at least in part because of the absence of a universal definition, the vagueness of its description, and the lack of observations in authentic context in which behavior is assessed [11–13].

According to the CanMEDS framework, a professional is “committed to the health and well-being of individuals and society through ethical practice, profession-led regulation and high personal standards of behavior” [14, 15]. The Dutch residency regulations [16] interpret the domain as follows: (1) The specialist provides care in an honest and involved manner and can justify his/her own actions. (2) The specialist displays adequate personal and interpersonal behavior. (3) The specialist knows the limits of his/her own competence and acts within them. (4) The specialist practices medicine according to the usual ethical standards of the profession and takes an active part in professional quality improvement. While the first sentence underscores principles such as honesty, involvement/compassion, transparency, justification, and accountability, the second sentence introduces behavior toward self and others. The third requires modesty and self-awareness. The last sentence introduces circular reasoning: professionals are those who act on and improve the standards of the profession.

While ethical and professional codes provide some direction in medical practice, these codes focus on physicians’ motives, intentions, and excellent behavior rather than specifying which behavior is unacceptable. Addressing unacceptable behavior in residency, which could ultimately lead to dismissal, is, however, important for improvement of patient safety, reducing liability, increasing employee satisfaction, and upholding institutional reputation [17–19]. Two psychiatry studies, conducted more than three decades ago, identified reasons for dismissing psychiatry residents using program director surveys. Roback and Crowder [20] found that 3.3% of the American psychiatry residents in training between 1981 and 1985 were dismissed, 64% due to unprofessional behavior. Of those residents, 17% displayed psychological disturbances; 22% had interpersonal problems with supervisors, other residents, or patients; and 25% had irresponsible, unethical, or illegal behavior. Russell et al. [21] found that in 1971, 1.9% of the dismissed American psychiatry residents were dismissed for unethical behavior. While these studies shed some light on the behavior required for the professional domain, no recent studies applied the CanMEDS professional domain to specify unprofessional behavior in psychiatry residency. In particular, little is known about specific unprofessional behavior that program directors consider sufficient cause for dismissing a resident, since such decisions are usually confidential.

The aim of this study was to specify what is considered unprofessional behavior by identifying the reasons of program directors to dismiss psychiatry residents. The following research questions were formulated: Which descriptions of behavior did the program directors use in the conciliation board procedure to justify a resident’s dismissal because of failure to meet the CanMEDS professional domain, and how extensive was the overlap with failures to meet other CanMEDS competencies?

## Methods

We performed a retrospective case study of conciliations at The Royal Dutch Medical Association (RGS KNMG) board regarding residency dismissal from 2011 to 2020. Every 3 months, the residency program director evaluates the resident’s performance and decides whether the resident is allowed to continue training and what focus is applied during each consecutive training phase. If the program director considers the resident unapt to continue training after careful deliberation, the director may decide to dismiss, usually after a formal intensive remediation and guidance program of at least 3 months and at max 6 months tailored to the needs of the resident. Regarding the program director’s decision to dismiss, the resident may request mediation from the centralized educators’ committee of the hospital, which is mandatory for each Dutch teaching hospital. In case of unsuccessful local mediation, the resident may subsequently request national conciliation from the board of The Royal Dutch Medical Association.

The RGS KNMG board is a national conciliation board with two legal professionals, a program director, and a resident from another institution. The conciliation board does not judge the aptitude for residency but considers whether the program director made a deliberate and careful decision. The conciliation board can decide to continue the training program, sometimes in another institution with additional intensive assessments. The conciliation board’s decision is binding to both parties. Nevertheless, the resident is entitled to continue legal action, although residents do so seldomly. The decisions of the conciliation board are anonymized and made publicly available in annual reports online [22]. The annual reports with all anonymized caselaw are available online as open-source data. Our research involved the analysis of existing data, already publicly available and anonymized by the conciliation board itself, ensuring that individual subjects cannot be identified.

The decisions we selected included both outcomes: whether the decision was in favor of the resident or the program director. Decisions with a favorable outcome for the resident were compared with decisions with an unfavorable outcome. Because of the lack of prior research among psychiatry residents and the availability of research on residents in family medicine requiring remediation [5], we included decisions in family medicine residents as a control group. This method enabled us to estimate potential differences between the research results of conciliations caselaw and regular residents requiring remediation. At least in part, competencies in psychiatry might be similar to some of the competencies in family medicine, as both specialties have combined rotations. The first author performed a systematic within-case analysis documenting general characteristics, such as the resident’s gender, year of residency, and sick leave. Thereafter, cross-case analysis builds an overall understanding of the caselaw on psychiatric residents as a group. Finally, the first author compared the psychiatry caselaw with the control group of family medicine caselaw on outcomes with Jamovi 2.2.5. with chi-square, Fisher-exact, and Student’s *t*-test (*p* < 0.01 was considered a significant difference).

## Results

The authors identified 24 cases of dismissal from training in psychiatry due to failures to meet CanMEDS competencies between 2011 and 2020. The conciliation board confirmed the program directors’ decision to dismiss in 63% (12 of 19) of the psychiatry cases. There were no differences in characteristics between cases decided in favor of the resident and cases decided in favor of the program director (*p* > 0.01 when compared regarding gender, year of residency, and number of insufficient competencies). However, of these 19 residents, only 4 were eventually registered as a psychiatrist. The others presumably failed another attempt to remediate. Most decisions literally described which CanMEDS competencies the resident had failed to meet. The authors literally cited these resident behaviors judged as unprofessional by the decision board and arranged them according to the principles laid down in the Canadian Medical Association Code of Ethics [23], which applies both to residents in psychiatry and to residents in family medicine. This code contains clear descriptions of principles, and there is no such code available in Dutch.

Insufficient professionalism was mentioned in 19 of the 24 cases (79%). The cases that were considered insufficient in professionalism were also considered insufficient in several other CanMEDS competency domains, most often communication, medical expertise, and/or management. The authors purposefully sampled 19 cases of training dismissal because of unprofessionalism in family medicine between 2011 and 2019 to match the number of psychiatry residents insufficient in professionalism. Characteristics of caselaw are presented in Table 1 and were not significantly different between groups. Half of the psychiatry residents with deficits in professionalism went on sick leave at some time. Six out of 19 (32%) psychiatry residents previously functioned without problems before an illness episode such as psychosis, depression, or burnout. Five out of 19 (26%) family medicine residents went on sick leave, but only one of them functioned without problems before.

**Table 1:** Characteristics of residents dismissed from training due to lack of professionalism (2011 to 2020)

Specialty	Psychiatry (*n* = 19)*		Family medicine (*n* = 19)	
Characteristic	%	#	%	#
Gender				
Male	52.6	10	52.6	10
Female	47.4	9	47.4	9
Sick leave				
Functioning well before sick leave	31.6	6	5.3	1
In total	47.4	9	26.3	5
	Mean	Range	Mean	Range
Nominal years of training	4.5	–	3	–
Years of training until dismissal	2.6	0.75–4	2.1	0.75–3
Number of insufficient domains	3.8	1–7	3.4	1–7

^*^In one of the cases, the resident was deficient in professionalism, but it is unclear from the case descriptions what the specific problems were

**Fig. 1:**
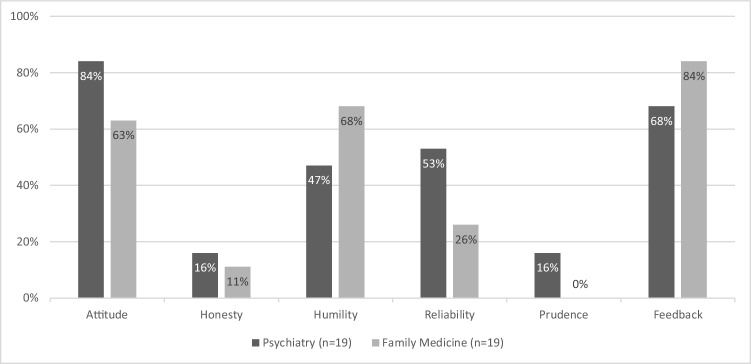
Failure to meet the professional domain. Note: Attitude = inadequate attitude toward patients, nurses, supervisors, conflicts with staff, or lack of compassion or commitment. Honesty = dishonesty. Humility = overstepping the limits of knowledge, not seeking help/advice/supervision at the appropriate time. Reliability = not keeping appointments with supervisors or teachers. Prudence = carelessness. Feedback = not being able to profit from feedback, or to follow advice

The cited behaviors of psychiatry residents dismissed for failure to meet the CanMEDS professional domain requirements are displayed in Table 2. The qualification and quantification of these behaviors are presented in Fig. 1 and compared with those of dismissed residents in family medicine (not significant *p* > 0.01). Most psychiatry residents were considered insufficient in their attitude (16 of 19, 84%) or in their ability to profit from feedback (13 of 19, 68%), followed by lack of reliability (10 of 19, 53%) or humility (9 of 19, 47%). Insufficiencies in honesty or prudence were least frequent in both groups of residents.

**Table 2:** Behavior indicating failure in professionalism in psychiatry (*n* = 18)

Major Theme	Subtheme Description
Attitude	
Patients	Trouble with establishing and maintaining contact with patients, inappropriate treatment of patients, including unintentional sexual intimidation
Nurses	Hostile posture toward nurses, making jokes about patients in team meetings
Peers	Insufficient collegiality
Supervisor	Troublesome teaching relationship
Institute	Rejection of evidence-based standards or institute regulations
Conflicts	Interaction problems, suspicion or frequent misunderstandings
Compassion	Lack of empathy, compassion or commitment to patient care
Honesty	Resumé fraud
	Taking patient medication home
	Concealing incidents
Humility	Overstepping the limits of one’s knowledge, blind spots in knowledge such as an inadequate sense of urgency, too little consultation of the supervisor, not seeking for help/advice/supervision within the appropriate time
Reliability	Not keeping appointments with supervisors or teachers, such as frequent absence on training days or disregard of deadlines
	Unavailability when on call
Prudence	Carelessness in the transmission and continuity of care, such as not writing letters after intake or discharge of care or not regularly keeping medical records
Feedback	Externalizing problems
	Questioning or not recognizing received feedback, or inability to profit from it
	Insufficient reflection on self or actions
	Being unteachable

**Fig. 2:**
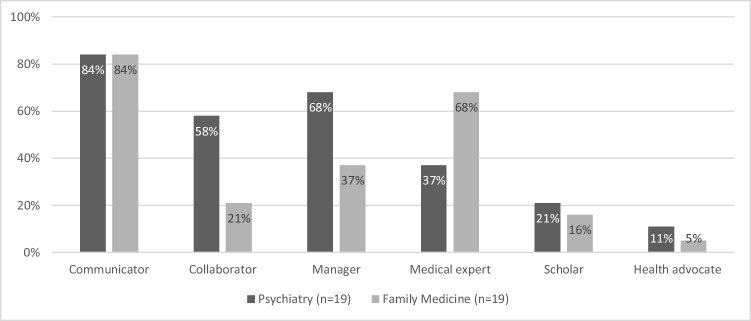
Deficiencies in CanMEDS competencies in residents dismissed due to unprofessionalism

Residents with deficits in professionalism also performed insufficiently regarding communication in 84% of the cases. This percentage was similar in psychiatry and family medicine. In psychiatry, more residents with deficits in professionalism were also considered insufficient in collaboration and management, whereas in family medicine, more residents with deficits in professionalism were considered insufficient in the domain of medical expert (both not significant *p* > 0.01). These results are presented in Fig. 2.

## Discussion

This study aimed to specify what is considered unprofessional behavior in residents by identifying program directors’ reasons for dismissing residents from the psychiatry training program in the Netherlands. A structured analysis of caselaw brought before the Dutch conciliation board revealed that insufficient professionalism in psychiatry residency was mostly due to problems in attitude or to inability to profit from feedback. Half of the residents lacked reliability or humility or both. There was considerable overlap between insufficiencies in professionalism and insufficiencies in communication, collaboration, and management.

The present study illustrates the nature of unprofessional behavior of poorly performing psychiatry residents in the CanMEDS era. Worldwide, there is no recent psychiatry research to which the present study can be compared. The authors tried to control for potential sampling bias, thus including only residents who appealed their dismissal and not all residents requiring remediation, using the caselaw of residents in family medicine as a control group. Our results of CanMEDS insufficiencies in the caselaw of residents from family medicine were in line with the insufficiencies mentioned by Vermeulen et al. 2016 [5] in their retrospective Dutch case study of a single family medicine institution. The latter suggests that conciliation board cases in family medicine have characteristics in common with poorly performing residents with remediation needs, which suggests that the potential selection bias influencing our results was limited.

In contrast to Roback and Crowder [20], who found that in 1989, 64% of the psychiatry residency dismissals were due to unprofessional behavior, the present study found a higher proportion of 79% (19 of the 24 cases). The difference between these findings may be due to the fact that Roback and Crowder [20] used program director surveys to identify reasons to dismiss psychiatry residents, rather than caselaw. A program directors’ survey will also include cases of misbehavior that are too obvious to be appealed. Among those dismissed for unprofessional behavior in our study, deficits in attitude were more frequent than in the study of Roback and Crowder [20] (16 of 19, 84%, versus 40 of 184, 22%), as were ethical problems, such as lack of reliability (10 of 19, 53%) or humility (9 of 19, 47%), and lack of honesty or prudence (both 3 of 19, 16%): Roback and Crowder [20] identified and categorized irresponsible behavior (failure to attend class, poor patient follow-up) in 31 of 184 cases (17%), unethical behavior (dating a patient, breach of confidentiality) in 8 of 184 cases (4%), and illegal behavior (inappropriate prescription of drugs) in 7 of 184 cases (4%). However, they did not provide complete descriptions of unprofessional behavior.

The higher rates of unprofessional behavior found in the present study, compared with Roback and Crowder [20], suggest that program directors, over time, might have become stricter in what they regard as unprofessional behavior. They may have set higher standards on residents’ attitudes, especially regarding their ability to deal respectfully and productively with feedback from patients, as well as colleagues and supervisors. This supposed change in strictness may be a response to a societal, public trend to become increasingly critical of doctors’ attitudes with which residents also have to learn to deal effectively [24] in their process of professional identity formation.

While the number of residents in the present study is small, strengths are its completeness, as it included all the decisions of the conciliation board on dismissed psychiatry residents in the Netherlands over a 10-year period, and the case control comparison with family medicine. These results provide a unique insight into the actual reasons for dismissal disputed by the resident, as the Dutch conciliation board allows public access to its anonymized decisions, whereas decisions regarding dismissal from the United States are confidential and hence not available for research. Another strength of the present study is that the descriptions of behavior captured in caselaw derived from resident portfolios are probably more reliable than the personal memories of program directors gathered in a survey.

In the Netherlands, skills pertaining to the CanMEDS competency domains are assessed during residency training. Unlike in the United States, there is no certification exam in the Netherlands; consequently, whether a resident can be registered as a psychiatrist entirely depends on the decision of the program director. The cases in this study represent approximately 1.4% (24 of 1664 [= 749/4.5 × 10 years) of all the Dutch psychiatry residents [25, 26]. Unfortunately, supplementary data on the number of psychiatry residents dismissed is unavailable in the Netherlands. However, the current study represented a higher percentage of dismissed residents than in a mixed specialty study from Canada [27], suggesting a relatively small selection bias in our sample. However, the lack of actual data on dismissed residents who do not appeal is a notable limitation of the current study.

Lack of professionalism is an important aspect in poorly functioning residents [4, 25] and should be considered relevant also in future assessments of entrusted professional activities. Since the inability to profit from feedback was mentioned in many of the caselaw included in this study (13 of 19, 68%), further research should elucidate the more detailed aspects of this particular competency. A focus group study with clinical supervisors and/or residents might be an informative addition to the descriptive caselaw study herein described. We hypothesize that residents who have difficulties with theory of mind, emotion perception, mentalization, and empathy might be impaired to profit from feedback as well as suffer from poor communication skills. There could also be a group of residents unable to respect authority, unable to trust the feedback givers’ intentions, or unable to differentiate performance feedback from personal appreciation. That group of residents might suffer from unadaptive character traits.

The program directors involved in our caselaw unsuccessfully used several remediation strategies for their residents. A successful remediation strategy is probably tailored to the specific needs of the resident. From this perspective, in some cases, a notice of deficiency in professional behavior could create a residents’ awareness, for example, by defining the desired behavior, timeline for improvement, and consequences of noncompliance to the desired behavior [28]. Conversations with a coach, mentor, or therapist about residents’ perceived barriers to appropriate behavior have been applied and may contribute to remediation. In addition, the program directors tended to assess more, while using workplace assessments, multisource feedback, sample assessments of patient files and letters, and direct observations of patient contacts, by several different clinical supervisors, peers, and colleagues. In some caselaw, the separation of assessment and clinical supervision was performed by appointing two different clinicians for these tasks. In other cases, the program directors changed either the residents’ workplace, institution, supervisor, or program director. This multitude of remediation strategies applied as evidenced by our caselaw analysis thus failed to successfully remediate these residents. Further effective evidence-based strategies to rectify unprofessionalism have yet to be elucidated.

An interesting finding that merits further investigation is that a considerable number of the dismissed residents went on sick leave before dismissal and that only a few performed well before their sick leave. Sickness resulting in sick leave might be a signal that a resident is unable to fulfill the expectations and responsibilities of residency training, whereas most literature on overpressure and burnout during residency focused on the educational climate [29] and regarded burnout as a cause of underperformance [30, 31]. The residents in our sample suffered from clinically relevant disabilities ranging from psychosis and burnout to attention disorder, mood disorder, and perhaps disturbances in social cognition. Mental disability can potentially interfere with a resident’s performance, probably in the domains of medical expertise, communication, and also in the professional domain [32]. Professional behavior requires self-monitoring, self-care, and recognizing one’s limitations of competence even during illness and due to illness. Mental conditions could impair those abilities and should be addressed if contributory to the displayed unprofessional behaviors. In our caselaw analysis, we found residents who persistently did not learn how to recognize their limitations, in spite of coaching, guidance, and patience from their clinical supervisors and program directors. More research is required on the causal relationship between sick leave and incapacity for residency training, since understanding this relationship may help prevent suffering among residents.

Another area for further research arises from the considerable overlap that was found between insufficiencies in professionalism and insufficiencies in communication, as well as in collaboration and management. Clear and respectful communication is recognized as an essential part of professionalism in other specialties [33]. The relevance of communication for psychiatry residents has been elucidated before, showing that communication is a specific form of medical expertise that is required to be able to connect with a patient and to comfort and confront patients with compassion and clarity [25]. Further research should clarify how overlapping domains co-occur in psychiatry and how they can be optimized in training. Aptitude for these domains might deserve extra attention during selection procedures. Therefore, selection procedures for postgraduate training positions may include tools to assess residents on professionalism, judgment, and communication, such as the multiple mini-interview [34], the situation judgment test [35], and other tools from industrial and organizational psychology [36], in addition to reference checking. However, none of these tools have sufficient positive and negative predictive power regarding a priori identification of *structural* patterns of either professionalism or unprofessionalism. Therefore, longitudinally assessing professional behavior will remain essential in the clinical workplace.

In these disputed cases of residents who challenged their program director’s decision, lack of professionalism was specified as deficits in attitude, violations of moral principles, and inability to profit from feedback. While unprofessional behavior in family medicine generally overlapped with deficiencies in medical expertise (thus in medical knowledge and medical skills), unprofessional behavior in psychiatry residency commonly overlapped with deficiencies in communication, collaboration, and management. The identified descriptions of unprofessional behavior can help to formulate the precise requirements of professional behavior for psychiatry residents more explicitly in selection criteria, training curricula, and remediation programs to safeguard the specialty’s high standards of professionalism.

## Declarations

The institutional review board (IRB) of Maastricht UMC + declared that the Medical Research Involving Human Subjects Act (WMO) does not apply to this study and official approval by the IRB is not required.
